# Necrostatin-1: a promising compound for neurological disorders

**DOI:** 10.3389/fncel.2024.1408364

**Published:** 2024-06-27

**Authors:** Ke-qian Chen, Shu-zhi Wang, Hai-bo Lei, Xiang Liu

**Affiliations:** ^1^Department of Clinical Pharmacy, Xiangtan Central Hospital, Xiangtan, China; ^2^Institute of Pharmacy and Pharmacology, School of Pharmaceutical Sciences, University of South China, Hengyang, China

**Keywords:** necroptosis, necrostatin-1, neurological disorders, apoptosis, necrostatins

## Abstract

Necrostatin-1, a small molecular alkaloid, was identified as an inhibitor of necroptosis in 2005. Investigating the fundamental mechanism of Necrostatin-1 and its role in various diseases is of great significance for scientific and clinical research. Accumulating evidence suggests that Necrostatin-1 plays a crucial role in numerous neurological disorders. This review aims to provide a comprehensive overview of the potential functions of Necrostatin-1 in various neurological disorders, offering valuable insights for future research.

## 1 Introduction

**Figure 1:**
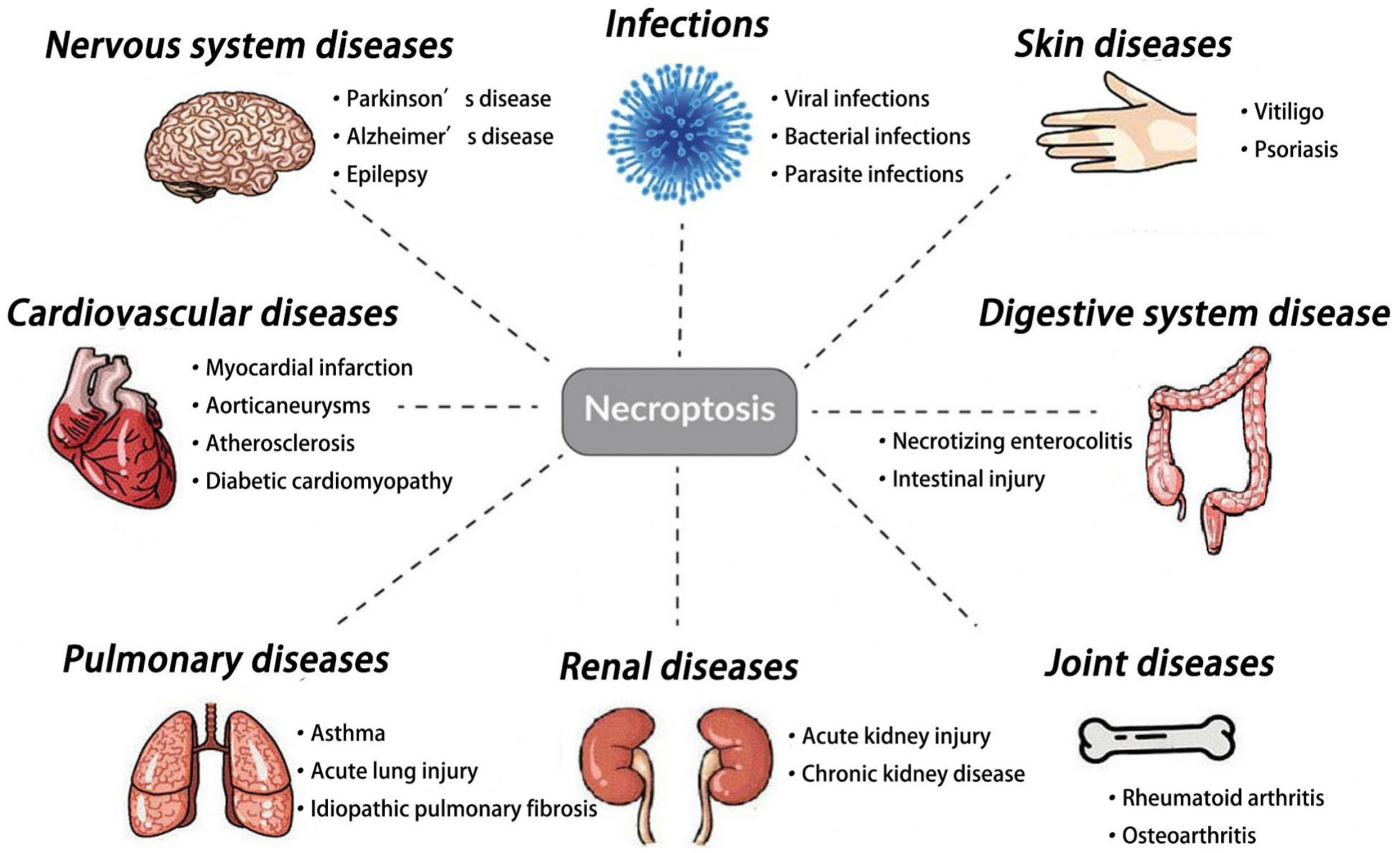
Main roles of necroptosis in various tissues.

In recent years, exploring the mechanisms of cell death has been a hot topic in medicine, cytology, and biology. Cell death can occur through various pathways, such as necrosis, apoptosis, necroptosis, pyroptosis, and ferroptosis (Yin et al., 2015). Necroptosis, a form of programmed cell death, has been shown to play a crucial role in immune regulation, tissue damage, and tumorigenesis (Gong et al., 2019; Gao W. et al., 2022) (Figure 1). Morphologically, necroptosis shares similarities with necrosis, characterized by cell swelling, organelle swelling, cell lysis, and the release of cellular debris (Davidovich et al., 2014). Necrostatins are a class of compounds that prevent necroptosis, including Necrostatin-1, necrostatin-2, necrostatin-5, and necrostatin-7 (Degterev et al., 2008). Since its discovery in 2005, Necrostatin-1 has become the most widely used necroptotic inhibitor (Degterev et al., 2005). Further studies have revealed that Necrostatin-1 specifically inhibits receptor-interacting protein 1 (RIP1). Geng et al. (2017) investigated the pharmacokinetics and bioavailability of Necrostatin-1 using an LC–MS/MS method, reporting an absolute bioavailability of 54.8%. Elucidating the fundamental mechanism of Necrostatin-1 and its role in various diseases is of great importance for both scientific and clinical research. Emerging evidence suggests that Necrostatin-1 possesses numerous pharmacological activities, including anti-cancer (Liu et al., 2015; Polito et al., 2016), anti-osteoporosis (Feng et al., 2018; Chen et al., 2018b; Feng et al., 2023), anti-glaucoma (Dong et al., 2012; Liu M. et al., 2022), anti-periodontitis (Yan et al., 2018; Tan et al., 2023), anti-osteoarthritis (Liang et al., 2018), and protective effects on the kidneys (Linkermann et al., 2013; Dong et al., 2018; Shen et al., 2019), lungs (Guan et al., 2017; Mou and Mou, 2020), liver (Zhou et al., 2013; Kim and Lee, 2017; Xie and Huang, 2019), heart (Carbone et al., 2016; Qiao et al., 2021; Erdogmus Ozgen et al., 2022), and nervous system and so on. Currently, increasing studies are exploring the neuroprotective role of Necrostatin-1 in neurological disorders. Therefore, the published work in this topic should not be neglected. Compared with other review papers (Zhang et al., 2017; Liao et al., 2020; Yu et al., 2021), this paper reviews the latest research of Necrostatin-1 in neurological disorders. Meanwhlie, this paper also introduces the “Toxicity of Necrostatin-1 in nervous system” and “Necrostatin-1 plays a neuroprotective role via other cell death pathways.” These findings will offer valuable insights for future research.

Necroptosis plays an important role in various organs, such as the bone, brain, heart, kidney, skin, lungs, colon and so on.

## 2 Signaling pathway of Necrostatin-1

**Figure 2:**
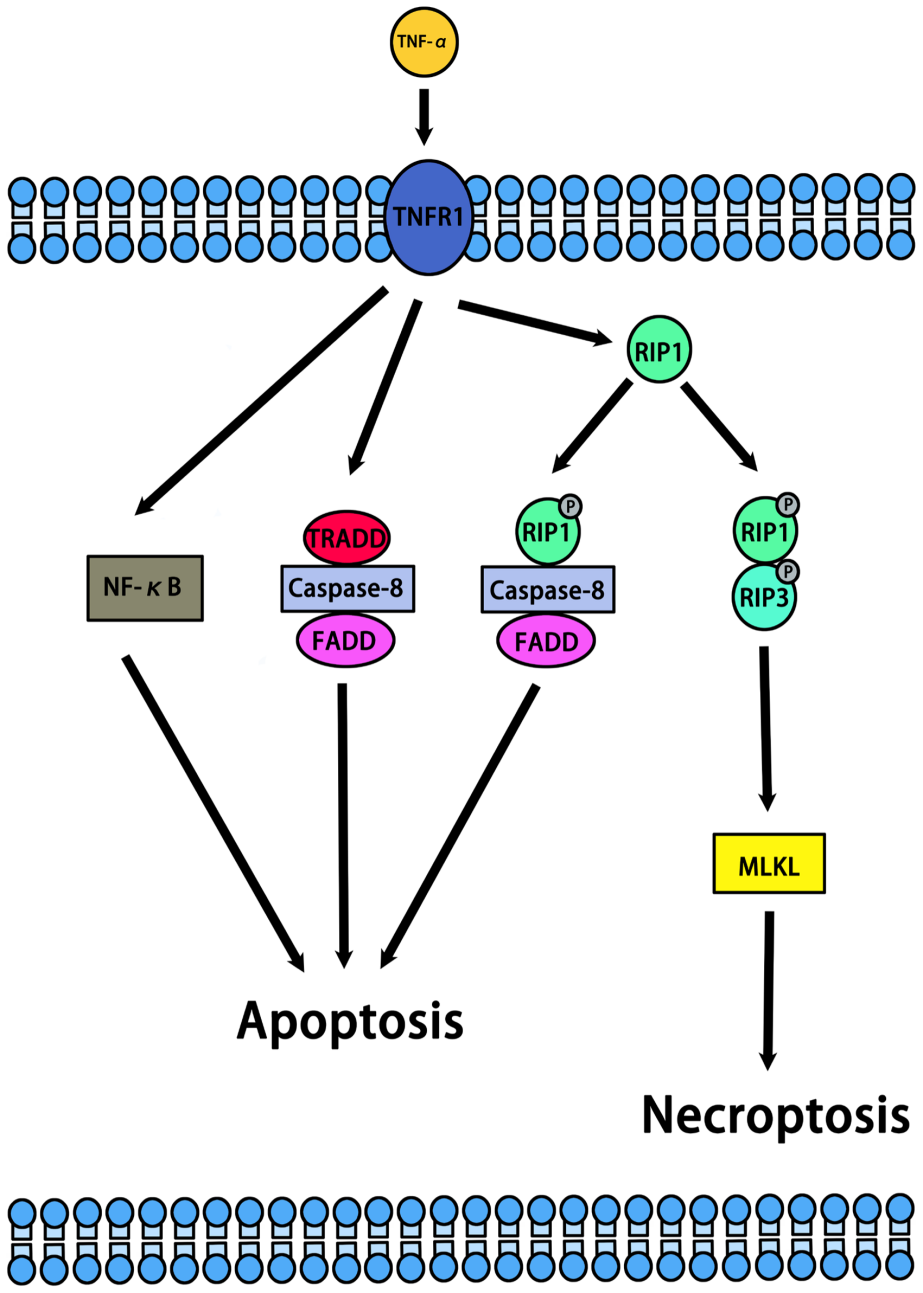
Molecular pathways of TNF-α induced necroptosis.

**Figure 3:**
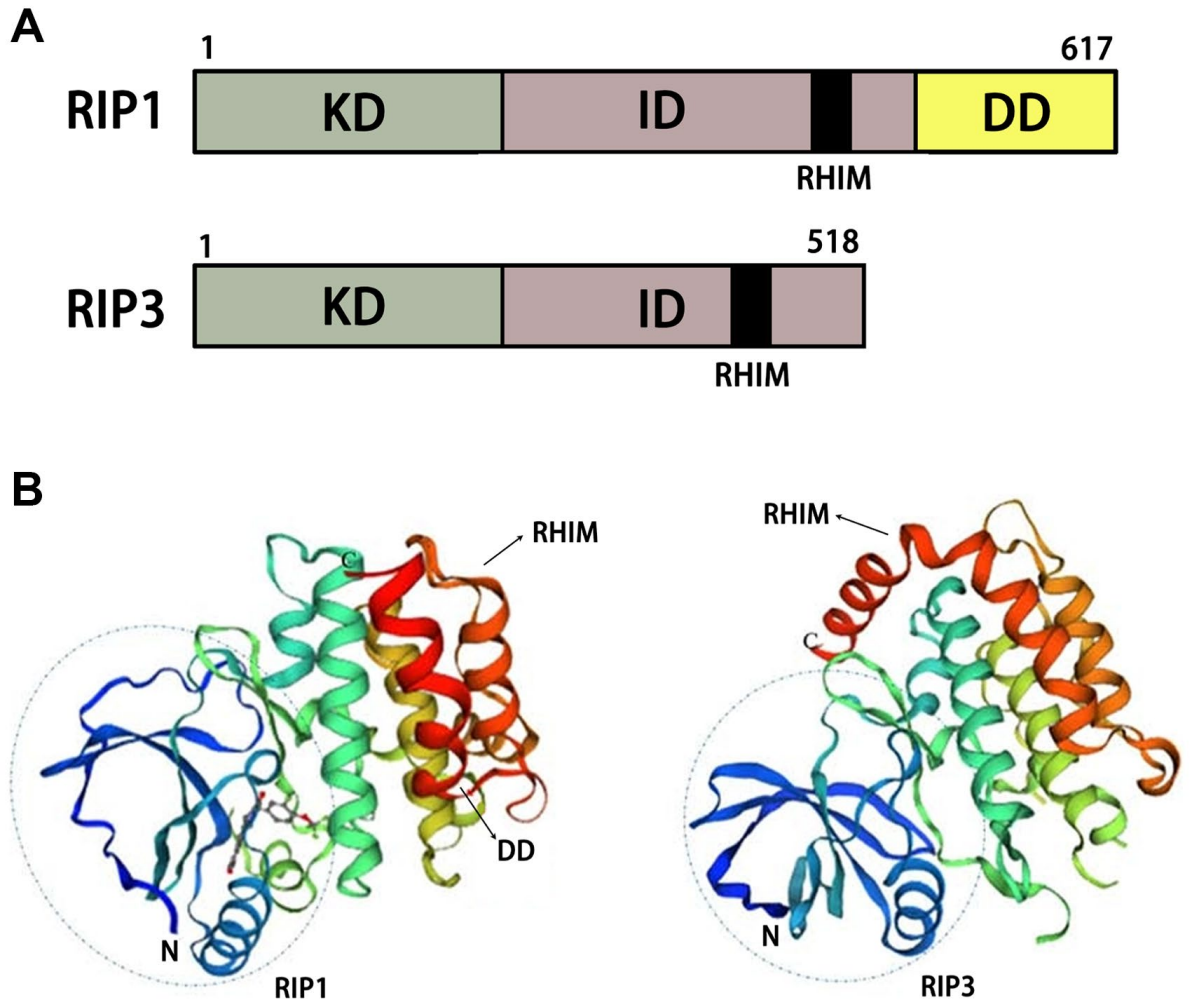
Structural diagrams of RIP1 and RIP3. **(A)** Schematic of functional domains of RIP1 and RIP3. **(B)** Protein tertiary structures of RIP1 and RIP3. KD, kinase domain; ID, intermediate domain; RHIM, RIP homotypic interaction motif; DD, death domain.

Necroptosis is a form of programmed necrosis that is independent of caspase regulation. When caspase is inhibited or not activated, necroptosis is activated (Zanetti and Weinlich, 2021). Previous studies have indicated that necrostatins are a class of compounds that inhibit RIP1. In normal and pathological conditions, necrostatins play an important role by inhibiting necroptosis or other pathways. In cells, necroptosis can be initiated by multiple upstream regulators, including TNF-α, FASL, APO-1 L, TRAIL, and IFN-α/β. Among them, TNF-α is the most important upstream regulator of necroptosis (Kearney et al., 2015; Pinci et al., 2022) (Figure 2). The binding of TNF-α to TNFR1 on the cell membrane stimulates different signaling pathways, including necroptosis, RIP1-dependent apoptosis (RDA), RIP1-independent apoptosis (RIA), and nuclear factor kappa B (NF-κB). Meanwhile, RIPK1, RIPK3, and MLKL are important downstream regulators of necroptosis. The mechanism of necroptosis is related to the activation of RIP1, RIP3 and MLKL (Cao and Mu, 2021) (Figure 3). By interacting with the T-loop, necrostatins can potently inhibit RIP1 autophosphorylation. RIP1 phosphorylation leads to the recruitment of RIP3 to RIP1 and subsequent formation of RIP1-RIP3 complex. This complex induces the phosphorylation of MLKL, which forms small holes in the plasma membrane. Eventually, disruptions of the plasma membrane lead to cell death (Cao and Mu, 2021). Therefore, necrostatins efficiently blocks RIP1/RIP3/MLKL signal transduction by inhibiting RIP1 phosphorylation. Interestingly, Necrostatin-1 has no direct inhibitory effect on RIP3 and does not block its autophosphorylation. In addition, necrostatins may be involved in hair cycle regulation under normal physiological conditions. Mechanistically, necrostatins upregulated Wnt3a and Wnt5b mRNA expression and increased the translocalization of β-catenin into the nucleus by stimulating β-catenin promoter binding activity (Zheng et al., 2020).

The combination of TNF-α and TNFR1 on the cell membrane stimulates different signaling pathways, including necroptosis, RIP1-dependent apoptosis (RDA), RIP1-independent apoptosis (RIA), nuclear factor kappa B (NF-κB). The RIP1 autophosphorylation sites include Ser14/15, Ser20, Ser161, and Ser166.

## 3 Necrostatin-1 and other cell death pathways

Increasing studies show that Necrostatin-1 not only suppresses necroptosis but also inhibits other cell death pathways (ferroptosis, apoptosis, pyroptosis). Ferroptosis is caused by the iron-mediated accumulation of lipid peroxidation, which is distinct from apoptosis and necroptosis (Newton et al., 2024). Necrostatin-1 not only perform a critical role in necroptosis but also in ferroptosis and maintain significant cellular mechanism. Yuk et al. (2021) demonstrated that Necrostatin-1 blocked ferroptosis through a mechanism independent from RIP1 and IDO inhibition in Huh7 and SK-HEP-1 cells. Caspase-8 is an executor of apoptosis. The aggregation of caspase-8 can lead to self-activation and activation of exogenous apoptotic pathways. Meanwhile, they promote the degradation of RIP1/RIP3 and lead to the closure of necroptosis signaling pathways (Fritsch et al., 2019). Some studies have explored the role of Necrostatin-1 on brain injury and its relationship with cell death pathways. They found that Necrostatin-1 not only blocked the occurrence of necroptosis but also significantly inhibited the expression of caspase-3 (an apoptosis-associated protein) and beclin-1 (an autophagy-associated protein) (Wang et al., 2012). In addition, Necrostatin-1 attenuates caspase-1-dependent pyroptosis induced by the RIP1/ZBP1 pathway in ventilator-induced lung injury (Shao et al., 2022).

## 4 Toxicity of Necrostatin-1

Although numerous studies have shown that Necrostatin-1 plays a neuroprotective role, there is evidence to support that Necrostatin-1 may damage the nervous system. In rotenone-induced PD model, Necrostatin-1 abolished necroptosis but did not prevent toxicity (Ye et al., 2023). Most likely, Necrostatin-1 activates a switch between cell death pathways. We think that Necrostatin-1 induces apoptosis and necroptosis by inhibiting mitophagy and promoting the accumulation of mitochondrial damage. Autophagy and necroptosis play an important role in most neurodegenerative diseases. Goodall et al. described a strong interaction between necrosome components and autophagy-related proteins. The knockdown of Necrostatin-1 abrogates this interaction and promotes apoptosis (Goodall et al., 2016). The inhibitory effect of Necrostatin-1 on autophagy has been reported in 6-hydroxydopamine treated neurons (Wu et al., 2015). Additionally, RIP1 knockdown upregulated autophagy, while Necrostatin-1 was shown to downregulate autophagy (Yonekawa et al., 2015). By inhibiting mitophagy, Necrostatin-1 affects mitochondrial morphology and mitochondrial clearance, which could enhance the effect of any Parkinsonian toxin (Alegre-Cortés et al., 2020). These different research results indicate that the underlying mechanism among Necrostatin-1, necroptosis and apoptosis is a complicated network, which is why Necrostatin-1 exhibits different effects in the nervous system.

## 5 Necrostatin-1 and inflammation

Neurodegenerative diseases are a large group of neurological disorders characterized by neuronal loss, including Alzheimer’s disease (AD), Parkinson’s disease (PD), and others (Dugger and Dickson, 2017). Although these neurodegenerative diseases have different pathogenetic mechanisms, inflammation plays a crucial role in their progression. Inflammation is the body’s defensive response to stimuli, and there is a mutually reinforcing effect between necroptosis and inflammation (Pasparakis and Vandenabeele, 2015). Necroptosis eventually leads to the release of cellular contents, causing an inflammatory response. Simultaneously, inflammation induces necroptosis via pro-inflammatory mediators (Kearney et al., 2015). Therefore, inhibiting necroptosis has great potential for treating neurodegenerative diseases by reducing inflammation. RIP1, a key target of necroptosis, promotes inflammatory responses via necroptotic cell death. In addition to inducing necroptotic cell death, RIP1 can also directly induce inflammation by producing pro-inflammatory cytokines, independent of cell death (Ofengeim and Yuan, 2013). As an inhibitor of RIP1, Necrostatin-1 exhibits significant anti-inflammatory effects in various inflammatory diseases, including hepatitis, pneumonia, and arthritis (Zhou et al., 2013; Jhun et al., 2019). Apoptosis of neutrophils is necessary for the resolution of inflammation. Necrostatin-1 is not only an inhibitor of necroptosis but also a promoter of neutrophil apoptosis, inhibiting the development of inflammation (Jie et al., 2016). Indoleamine 2,3-dioxygenase (IDO), a rate-limiting enzyme of tryptophan catabolism, plays a crucial role in inflammation. Necrostatin-1 is also an inhibitor of IDO (Vandenabeele et al., 2013), suppressing inflammation through this mechanism in addition to necroptosis inhibition. Neuroinflammation is responsible for generating and sustaining the sensitization of nociceptive neurons that lead to chronic pain. Liang et al. found that Necrostatin-1 ameliorates neuropathic pain by inhibiting neuroinflammation (Liang et al., 2019).

## 6 Necrostatin-1 and reactive oxygen species

Reactive oxygen species (ROS), highly reactive chemical substances, have long been studied in nervous system diseases (Singh et al., 2019). ROS, as regulators of mitochondrial dynamics, regulate neuronal development and function. However, a dramatic increase in ROS levels leads to cell structure damage under harmful conditions (Singh et al., 2019). Relevant studies indicate that the generation of ROS is probably RIP1-dependent (Jantas and Lasoń, 2021). ROS can increase the expression of RIP1/RIP3 and improve the stability of the RIP1-RIP3 complex (Chauhan et al., 2017). Glutamate, an important neurotransmitter, plays a crucial role in various neurological diseases. In HT-22 cells, Necrostatin-1 inhibits glutamate-induced oxytosis by increasing cellular glutathione (GSH) and reducing ROS (Xu et al., 2007). Additionally, Necrostatin-1 suppresses the phosphorylation of ERK1 and ERK2 after glutamate treatment (Zhang et al., 2013). CoCl2-induced neurotoxicity is associated with ERK1/2 phosphorylation and ROS production, which inhibit cell differentiation and lead to cell death. Chen R. et al. (2018) found that Necrostatin-1 inhibits CoCl2-induced neurotoxicity by decreasing ROS production and ERK1/2 phosphorylation. In H_2_O_2_-induced SH-SY5Y cell lines, Necrostatin-1 reduces oxidative stress-induced cell damage by inhibiting cathepsin D (Jantas et al., 2020). In peripheral nerve injury (PNI) and spinal cord injury (SCI) rat models, Necrostatin-1 can reduce ROS and inflammation (Yu et al., 2023). Further studies indicate that Necrostatin-1 not only inhibits necrosis by inhibiting RIP1/RIP3/MLKL but also inhibits apoptosis by activating Bcl-2 (Wang et al., 2014).

## 7 Necrostatin-1 and neurological disorders

### 7.1 Ischemic stroke and ischemia/reperfusion

Ischemic stroke (IS) often results in injury to oligodendroglia. Oligodendrocyte precursor cells (OPCs) are more vulnerable to cerebral ischemia than other mature oligodendroglia. Necrostatin-1 significantly promotes oligodendrocyte precursor cell survival and reduces white matter damage after cerebral ischemia (Chen et al., 2018a) through the RIPK1/RIPK3/MLKL signaling pathway (Deng et al., 2019). Necrostatin-1 also provides neuroprotection in neonatal hypoxia-ischemia (HI) by preserving mitochondrial function (Chavez-Valdez et al., 2012). Cerebral ischemia/reperfusion (I/R) induces selective neuronal injury in the CA1 region of the hippocampus. In cerebral I/R rats, Necrostatin-1 improves locomotive ability and relieves anxious behavior while decreasing the death rate of neurons through the RIP3/DAXX signaling pathway (Yang et al., 2017). Traumatic brain injury (TBI) is a leading cause of cerebral I/R injury. In a TBI mouse model, You et al. found that Necrostatin-1 has anti-inflammatory effects (You et al., 2008), while Wang et al. found that Necrostatin-1 inhibits autophagy and apoptosis (Wang et al., 2012). These results suggest that Necrostatin-1 may have therapeutic potential for IS and cerebral I/R.

### 7.2 Parkinson’s disease

PD is a neurodegenerative disorder characterized by the loss of dopaminergic neurons in the substantia nigra pars compacta. Several types of cell death, including apoptosis, autophagy-induced cell death, and necrosis, have been implicated in PD progression. In PD models, Necrostatin-1 prevents rotenone-induced necroptosis by affecting mitochondrial morphology (Alegre-Cortés et al., 2020) and exerts a protective effect on dopaminergic neurons by decreasing the expression of cathepsin B and increasing the expression of Bcl-2 (Wu et al., 2015; Jantas and Lasoń, 2022).

### 7.3 Epilepsy

Epilepsy is a common, highly debilitating neurological disease characterized by the abnormal discharge of brain neurons. Necrosis and apoptosis are the major forms of neuronal death post-epilepsy. In an epileptic mouse model, Necrostatin-1 significantly decreases damage to hippocampal tissue and downregulates apoptosis/necroptosis-related proteins such as cleaved-caspase-3, Bax, RIP1, RIP3, and MLKL (Lin et al., 2020). A 40 μM concentration of Necrostatin-1 has an optimal effect (Lin et al., 2020), and inhibition of necroptosis may prolong seizure latency (Guan et al., 2021).

### 7.4 Alzheimer’s disease

Aluminum (Al) is a risk factor for AD. In the Al-induced AD model, Necrostatin-1 enhances acetylcholine (ACh) levels and downregulates the expression of AD-related genes and proteins (Gao X. et al., 2022). Furthermore, Necrostatin-1 inhibits neural cell degeneration and alleviates learning and memory deficits (Qinli et al., 2013; Jantas and Lasoń, 2022). Postoperative cognitive dysfunction (POCD) has become a prevalent complication in the elderly population. It is particularly concerning that persistent POCD is likely to progress into AD. In POCD patients, sevoflurane stimulates calcium overload and neurotoxicity (Yin et al., 2022). Necrostatin-1 attenuated sevoflurane-induced cognitive impairment via brain-derived neurotrophic factor (BDNF)-tyrosine receptor kinase B (TrkB) signaling (Yin et al., 2022). Additionally, Necrostatin-1 mitigated cognitive dysfunction in prediabetic rats (Jinawong et al., 2020).

### 7.5 Subarachnoid hemorrhage

Cerebral vasospasm, cerebral edema, and blood–brain barrier disruption are pathogenic factors in subarachnoid hemorrhage (SAH). Relevant studies indicate that inflammation plays a crucial role in cerebral vasospasm. Sahin et al. found that Necrostatin-1 ameliorates SAH-induced vasospasm in a rat model (Sahin et al., 2021). Liu C. et al. (2022) discovered that Necrostatin-1 decreases inflammatory markers after SAH. In SAH rats, Necrostatin-1 also exerts a neuroprotective effect by attenuating blood–brain barrier disruption and brain edema (Su et al., 2015; Chen et al., 2019). Mechanistically, necroptosis is a significant cause of cell death after SAH. Necrostatin-1 attenuates early brain injury after SAH by inhibiting necroptosis (Chen et al., 2017; Jantas and Lasoń, 2022). Another study suggested that Necrostatin-1 plays a neuroprotective role by inhibiting apoptosis and autophagy pathways in the SAH model (Chang et al., 2014).

### 7.6 Spinal cord injury

SCI is a severe nerve injury. Endoplasmic reticulum stress (ERS) is a critical pathological consequence of SCI. Necrostatin-1 has a protective effect on the endoplasmic reticulum by inhibiting the expression of ERS-related genes and proteins, such as C/EBP homologous protein (CHOP), immunoglobulin-binding protein (BiP/GRP78), and X-box-binding protein-1 (XBP-1) (Wang et al., 2017). Moreover, Necrostatin-1 improves mitochondrial functions in SCI (Jantas and Lasoń, 2022). It decreases Ca^2+^ concentration, increases adenosine triphosphate (ATP) generation, inhibits cytochrome c release, and preserves the mitochondrial membrane potential (MMP) level (Wang et al., 2015). In SCI mice, Necrostatin-1 significantly promotes locomotor function recovery by inhibiting the M1 polarization of microglia/macrophages (Tang et al., 2021). Necrostatin-1 also attenuates experimental autoimmune encephalomyelitis (EAE) and delayed paraplegia after SCI (Wang et al., 2019; Nishijima et al., 2023).

## 8 Other RIP inhibitors in neurological disorders

**Figure 4:**
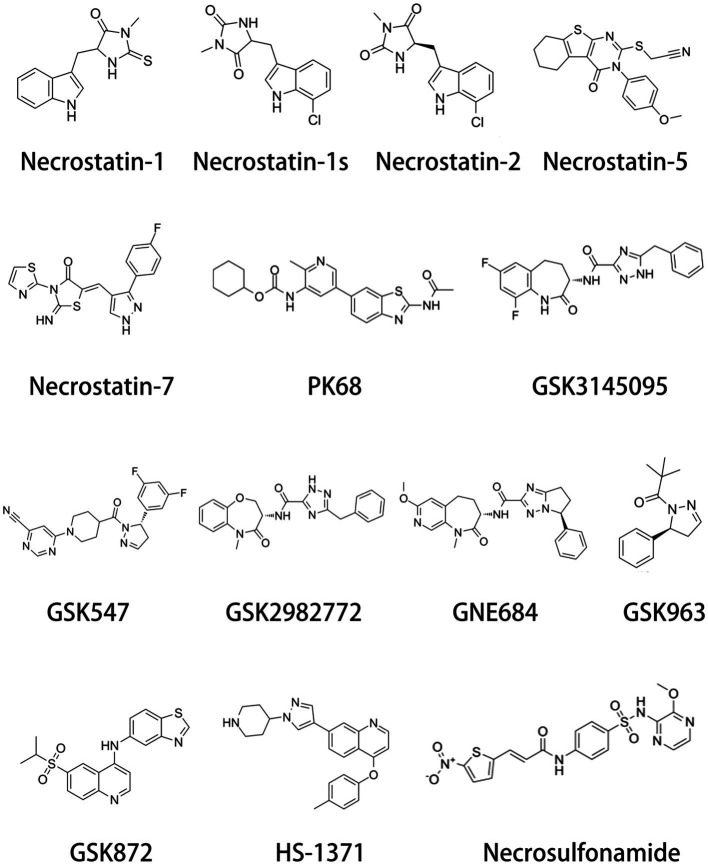
Chemical structure of necroptosis inhibitors. Necroptosis inhibitors include RIP1 inhibitors, RIP3 inhibitors, and MLKL inhibitors.

Increasing evidence suggest that RIP inhibitors play an important role in neurological pathologies. Necroptosis-associated RIP inhibitors include RIP1 inhibitors and RIP3 inhibitors (Figure 4 and Table 1). Besides the Necrostatin-1, Necrostatin-1 s is another important RIP1 inhibitor. Preeti et al. (2023) want to evaluate the neuroprotective effect of Necrostatin-1 s in the type-2 diabetes mellitus model. They found that Necrostatin-1 s mitigates cognitive decrement. Further, Necrostatin-1 s reduced tau and amyloid oligomer load. In the periventricular leukomalacia model, the expression level of RIP1 was drastically increased. Necrostatin-1 s greatly ameliorated cerebral ischemic injury and long-term neurobehavioral abnormalities, exhibiting a reduction of cerebral infarct size and neuronal loss (Sun et al., 2024). In addition, Kartik et al. (2023) found that Necrostatin-1 s significantly improve the survival of dopaminergic neurons in the PD mouse model. Other RIP1 inhibitors such as GSK772, PK68, GSK095, and GSK547 were not reported to improve nerve damage. GSK872 is a widely used RIPK3 inhibitor. Similar to Necrostatin-1 s, GSK872 improves various nerve damage such as retinal neuroinflammation, neurodegeneration, SCI, hydrocephalus and so on (He et al., 2021; Liu et al., 2021; Huang et al., 2023). Necrosulfonamide is a specific MLKL inhibitor. In a transient middle cerebral artery occlusion (tMCAO) rat model, necrosulfonamide reduces infarction volume and improves neurological deficits (Zhou et al., 2023). Besides the neuroprotective effects of tMCAO, necrosulfonamide also ameliorates SCI and intracerebral hemorrhage injury (Wang et al., 2018; Zhang et al., 2022). Interestingly, necrosulfonamide increased cleaved PARP-1 levels, indicating the protective effects of necrosulfonamide is not related to apoptosis (Zhou et al., 2017).

**Table 1:** Inhibitors of necroptosis.

Name	Target	CAS number	Molecular formula
Necrostatin-1	RIP1	4,311-88-0	C_13_H_13_N_3_OS
Necrostatin-1 s	RIP1	852,391–15-2	C_13_H_12_ClN_3_O_2_
Necrostatin-2	RIP1	852,391–19-6	C_13_H_12_ClN_3_O_2_
Necrostatin-5	RIP1	337,349–54-9	C_19_H_17_N_3_O_2_S_2_
Necrostatin-7	RIP1	351,062–08-3	C_16_H_10_FN_5_OS_2_
PK68	RIP1	2,173,556–69-7	C_22_H_24_N_4_O_3_S
GSK3145095	RIP1	1,622,849–43-7	C_20_H_17_F_2_N_5_O_2_
GSK547	RIP1	2,226,735–55-1	C_20_H_18_F_2_N_6_O
GSK2982772	RIP1	1,622,848–92-3	C_20_H_19_N_5_O_3_
GNE684	RIP1	2,438,637–64-8	C_23_H_24_N_6_O_3_
GSK963	RIP1	2049868-46-2	C_14_H_18_N_2_O
GSK872	RIP3	1346546-69-7	C_19_H_17_N_3_O_2_S_2_
HS-1371	RIP3	2158197-70-5	C_24_H_24_N_4_O
Necrosulfonamide	MLKL	1,360,614-48-7	C_18_H_15_N_5_O_6_S_2_

## 9 Application of Necrostatin-1

Beyond treating various diseases, Necrostatin-1 plays a crucial role in plastic surgery, preservation, transplantation, and inhibition of drug toxicity. Plastic surgery failure is a challenge for the medical cosmetology industry. Increasing research shows that Necrostatin-1 can treat various I/R injuries, such as those affecting the heart, lung, kidney, and skeletal muscle. In flap surgery, I/R injury is considered the primary problem. Liu et al. (2019) found that Necrostatin-1 has a protective effect against I/R injury in a skin flap model. These results suggest that Necrostatin-1 could be a promising novel strategy in plastic surgery. Cryopreservation of spermatogonial stem cells (SSCs) is important for preserving the lineages of valuable livestock and producing transgenic animals. As a potential cryoprotectant, Necrostatin-1 improves the cryopreservation efficiency of SSCs (Jung et al., 2020). Jo et al. (2015) also found that Necrostatin-1 improves the survival of mouse oocytes. Numerous studies show that Necrostatin-1 promotes the maturation, development, and graft function of neonatal porcine islets (Lau et al., 2020a,b, 2021), providing an effective strategy for the future application of islet grafts (Qin et al., 2022). Emerging evidence suggests that Necrostatin-1 has potential radical scavenging activities (Ushijima and Monzaki, 2023). Ning et al. found that Necrostatin-1 can decrease cisplatin-induced nephrotoxicity by inhibiting oxidative stress (Ning et al., 2018). Takemoto et al. discovered that Necrostatin-1 ameliorates acetaminophen-induced hepatotoxicity by inhibiting ROS (Takemoto et al., 2014). These results suggest that Necrostatin-1 has some benefit in alleviating drug toxicity. Interestingly, Necrostatin-1 can mitigate and treat radiation-induced damage in mice (Huang et al., 2016).

## 10 Discussion

In this review, we explored the mechanisms and roles of Necrostatin-1 in various neurological disorders (Table 2). Meanwhile, we propose that Necrostatin-1 has great clinical potential in the treatment of these disorders. In addition to treating various diseases, Necrostatin-1 plays an important role in plastic surgery, preservation, transplantation, and inhibition of drug toxicity. Nevertheless, there are still many questions regarding Necrostatin-1 that need to be addressed. First, Necrostatin-1 has a short half-life, which may affect its application. Second, it remains unclear whether Necrostatin-1 can affect one or multiple RIP1-dependent pathways in various neurological disorders. These findings suggest that the mechanism of Necrostatin-1 in disease is quite complex. In the future, it is necessary for scientists to further explore Necrostatin-1.

**Table 2:** Role of Necrostatin-1 in neurological disorders.

Subjects	Pharmacologic action	Function	Ref.
Male SD rats	Anti-neuropathic pain	RIP1↓ RIP3↓ TNF-α↓ IL-1β↓ Substance P↓	Liang et al. (2019)
Male ICR mice	Anti-ischemic stroke	RIP1↓ RIP3↓ MLKL↓ White matter damage↓	Chen et al. (2018a)
C57B6 mice	Anti-neonatal hypoxia-ischemia	NO↓ iNOS↓ Glutathione oxidation↓ HIF1-a↓ BNIP3↓	Chavez-Valdez et al. (2012)
Male SD rats	Anti-cerebral ischemia/reperfusion	RIP1↓ RIP3↓ Memory deficit↓ Cognitive impairment↓	Yang et al. (2017)
Mice	Anti-traumatic brain injury	Brain tissue damage↓ Cellular neuroinflammation↓	You et al. (2008)
Male CD1 mice	Anti-traumatic brain injury	Beclin-1↓ LC3-II ↓ Bcl-2↓ Caspase-3↓	Wang et al. (2012)
Healthy subjects Patients with two forms of PD	Anti-parkinson’s disease	TOMM20↑ PHB1↑ Mitochondrial morphology↓	Alegre-Cortés et al. (2020)
PC12 cells	Anti-parkinson’s disease	Cathepsin B↓ Bcl-2↑	Wu et al. (2015)
Male ICR mice	Anti-epilepsy	Cleaved-Caspase-3↓ Bax↓ RIP1↓ RIP3↓	Lin et al. (2020)
Male C57BL/6 mice	Anti-epilepsy	TNF-α↓ IL-1β↓	Guan et al. (2021)
Adult zebrafsh	Anti-alzheimer’s disease	Ach↑ RIP1↓ RIP3↓ PARP2↓ Bmf1↓ Rab25↓	Gao X. et al. (2022)
Murine cortical cells	Anti-alzheimer’s disease	Neural cell death↓ Cell viability↑	Qinli et al. (2013)
Male SD rats	Anti-postoperative cognitive dysfunction	RIP1↓ RIP3↓ MLKL↓ Cognitive impairment↓	Yin et al. (2022)
Male rats	Anti-cognitive dysfunction	NFκB↓ RIP1↓ RIP3↓ MLKL↓ Cleaved-Caspase-3↓	Jinawong et al. (2020)
Male Wistar albino rats	Anti-subarachnoid hemorrhage	Vasospasm↓	Sahin et al. (2021)
Male C57BL/6 mice	Anti-subarachnoid hemorrhage	TNF-α↓ IL-1β↓ IL-6↓	Liu C. et al. (2022)
Male ICR mice	Anti-subarachnoid hemorrhage	TNF-α↓ IL-1β↓ Necrotic cell death↓	Su et al. (2015)
Male SD rats	Anti-subarachnoid hemorrhage	TNF-α↓ IL-1β↓ IL-6↓ RIP3↓ MLKL↓ MMP-9↓	Chen et al. (2019)
Male SD rats	Anti-spinal cord injury	CHOP↓ BiP/GRP78↓ XBP-1↓	Wang et al. (2017)
Male SD rats	Anti-spinal cord injury	Ca^2+^↓ Cytochrome c↓ ATP↑	Wang et al. (2015)
Male C57BL/6 mice	Anti-spinal cord injury	Locomotor function recovery↑	Tang et al. (2021)
Female C57BL/6 mice	Anti-experimental autoimmune encephalomyelitis	IFN-γ↓ TNF-α↓ IL-1β↓ ROS↓ MMP↑	Wang et al. (2019)
Domesticated rabbits	Anti-delayed paraplegia	RIP1↓ RIP3↓	Nishijima et al. (2023)

## Author contributions

K-qC: Writing – original draft. S-zW: Writing – review & editing, Investigation. H-bL: Writing – review & editing, Formal analysis. XL: Writing – review & editing, Data curation.

## Glossary

RIP1: Receptor-interacting protein 1
RIP3: Receptor-interacting protein 3
TNF-α: Tumor necrosis factor-α
TNFR1: Tumor necrosis factor receptor 1
RDA: RIP1-dependent apoptosis
RIA: RIP1-indipendent apoptosis
NF-κB: Nuclear factor kappa B
MLKL: Mixed lineage kinase domain-like
AD: Alzheimer’s disease
PD: Parkinson’s disease
IDO: Indoleamine 2,3-dioxygenase
ROS: Reactive oxygen species
GSH: Glutathione
PNI: Peripheral nerve injury
SCI: Spinal cord injury
IS: Ischemic stroke
OPCs: Oligodendrocyte precursor cells
HI: Hypoxia-ischemi
LC–MS/MS: Liquid chromatography–mass spectrometry
I/R: Ischemia/reperfusion
TBI: Traumatic brain injury
Al: Aluminum
POCD: Postoperative cognitive dysfunction
SAH: Subarachnoid hemorrhage
ERS: Endoplasmic reticulum stress
CHOP: C/EBP homologous protein
XBP-1: X box-binding protein-1
ATP: Adenosine triphosphate
MMP: Mitochondrial membrane potential
EAE: Experimental autoimmune encephalomyelitis
SSCs: Spermatogonial stem cells
